# Medial and lateral hamstrings and quadriceps co-activation affects knee joint kinematics and ACL elongation: a pilot study

**DOI:** 10.1186/s12891-015-0804-y

**Published:** 2015-11-12

**Authors:** Benjamin G. Serpell, Jennie M. Scarvell, Mark R. Pickering, Nick B. Ball, Phillip Newman, Diana Perriman, John Warmenhoven, Paul N. Smith

**Affiliations:** Trauma and Orthopaedic Research Unit, Canberra Hospital, Woden, ACT Australia; Medical School, The Australian National University, Canberra, ACT Australia; Faculty of Health, University of Canberra, Bruce, ACT Australia; School of Engineering and Information Technology, University of New South Wales, Canberra, ACT Australia; Research Institute for Sport and Exercise, University of Canberra, Bruce, ACT Australia

**Keywords:** ACL, Anterior cruciate ligament, Muscle activation

## Abstract

**Background:**

Many injury prevention and rehabilitation programs aim to train hamstring and quadriceps co-activation to constrain excessive anterior tibial translation and protect the anterior cruciate ligament (ACL) from injury. However, despite strong clinical belief in its efficacy, primary evidence supporting training co-activation of the hamstrings and quadriceps muscles for ACL injury prevention and rehabilitation is quite limited. Therefore, the purpose of the study presented in this paper was to determine if hamstring-quadriceps co-activation alters knee joint kinematics, and also establish if it affects ACL elongation.

**Methods:**

A computed tomography (CT) scan from each participant’s dominant leg was acquired prior to performing two step-ups under fluoroscopy: one with ‘natural’ hamstring-quadriceps co-activation, one with deliberate co-activation. Electromyography was used to confirm increased motor unit recruitment. The CT scan was registered to fluoroscopy for 4-D modeling, and knee joint kinematics subsequently measured. Anterior cruciate ligament attachments were mapped to the 4-D models and its length was assumed from the distance between attachments. Anterior cruciate ligament elongation was derived from the change in distance between those points as they moved relative to each other.

**Results:**

Reduced ACL elongation as well as knee joint rotation, abduction, translation, and distraction was observed for the step up with increased co-activation. A relationship was shown to exist for change in ACL length with knee abduction (*r* = 0.91; *p* ≤ 0.001), with distraction (*r* = −0.70; *p* = 0.02 for relationship with compression), and with anterior tibial translation (*r* = 0.52; *p* = 0.01). However, ACL elongation was not associated with internal rotation or medial translation. Medial hamstring-quadriceps co-activation was associated with a shorter ACL (*r* = −0.71; *p* = 0.01), and lateral hamstring-quadriceps co-activation was related to ACL elongation (*r* = 0.46; *p* = 0.05).

**Conclusion:**

Net co-activation of the hamstrings and quadriceps muscles will likely reduce ACL elongation provided that the proportion of medial hamstring-quadriceps co-activation exceeds lateral.

## Background

Excessive tibial translation has been implicated as the cause of serious knee injuries such as anterior cruciate ligament (ACL) injury [1]. Therefore, the focus of many injury prevention and rehabilitation programs is to train co-activation of the hamstrings and quadriceps to constrain this [2, 3]. However, primary evidence supporting the role of hamstring-quadriceps co-activation for constraining tibial translation and subsequent protection of the ACL from injury is limited. This absence of evidence in spite of strong clinical belief in the efficacy of co-activation is likely due to the difficulty of measuring *in-vivo* tibial translation or ACL elongation while performing a dynamic task.

Tibial translation is typically ‘quantified’ by measuring passive or active knee joint laxity. Passive laxity is the ‘amount’ of passive motion observed in any plane or rotation prior to plateauing of a displacement tension curve [4]. Active laxity is the secondary motion observed in a plane or rotation during active movement which is not associated with the primary movement [4]. For example, some tibial translation may be observed when performing a step-up; the primary movement is knee extension and tibial translation the secondary. Passive knee joint laxity is typically measured *in-vivo* with anterior draw tests using knee arthrometers or manual tests such as Lachman’s test [1]. However, measures of passive laxity do not reflect functional instability as they are unable to evaluate the effect of muscular control. Active laxity has been implied from *in-vitro* cadaveric studies [5], however these studies still fail to evaluate the true effect of muscular influences [5]. More recently an *in-vivo* study which used fluoroscopy and electromyography (EMG) attempted to explain anterior tibial translation (ATT) and the role of hamstring-quadriceps co-activation in an ACL deficient population during both open and closed kinetic chain tasks (seated knee extension and step up respectively) [2]. However, the findings from that study are not conclusive since the EMG and fluoroscopy were not conducted concurrently and ATT was assumed from measuring patella tendon angle [2].

Recent advances in image registration techniques offer the possibility of real-time *in-vivo* measurement of ATT while executing dynamic tasks whereby computed tomography (CT) images are registered with fluoroscopy (video x-ray) to allow 4-D motion analysis of bone [6–8]. This methodology provides the opportunity for measuring kinematics with previously unachievable precision while concurrently measuring hamstring and quadriceps activity. Furthermore, by using a biomechanical model to locate the ACL attachments, measurement of the distance between those attachments can provide some insight into ACL length and tension. However, such a procedure is financially costly and requires some ethical consideration due to the radiation dosage administered. Therefore, pilot research using this technique is required to establish its ‘value’.

This pilot study had two primary aims; first, to establish if co-activation of hamstring and quadriceps muscles altered knee joint motion during a step-up task, and secondly to examine if ACL elongation (maximum change in distance between the ACL attachments) was related to co-activation of the hamstring-quadriceps muscles during a step-up. We hypothesized that co-activation of the hamstrings and quadriceps would constrain the knee in terms of rotation and translation and reduce the ACL elongation when performing a step-up.

## Methods

### Experimental approach

This was a descriptive cohort study of healthy males from a single professional rugby union club. A CT scan of each participant’s dominant knee was acquired. Participants then performed two step-ups in view of the image intensifier of a fluoroscopy machine. The first step up was performed with a low level of co-activation; that is, participants stepped up onto a box as they typically would step-up onto a box or walk up a step. Prior to the second step-up, participants were taught how step-up with deliberate co-activation of their quadriceps and hamstring muscles. Muscle activity was recorded with EMG to confirm the increase in co-activation on the second step-up. The CT scan and fluoroscopy images were image-registered to enable kinematic analysis of knee rotations and translations as well as modelling of ACL length by mapping the distances between the bony attachment sites.

A step-up task was used in order to be consistent with previously published studies [2], and because tibial translation was more likely to be seen during a closed kinetic chain task as opposed to an open chain task. Only one repetition of each step-up was performed under fluoroscopy to keep radiation dose within ethical limits.

### Participants

Five males all from a single professional rugby union club aged 24.9 ± 4.1 years, height 184.8 ± 9.1 cm and weight 90.1 ± 16.3 kg (mean ± SD). All had ACL intact knees and were free of lower limb injury.

### Procedures

Each participant gave written informed consent according to institutional ethics approval for this study prior to participating. Ethical approval to conduct the research was granted by the ACT Health human research ethics committee and also the Australian National University human research ethics committee.

**Fig. 1:**
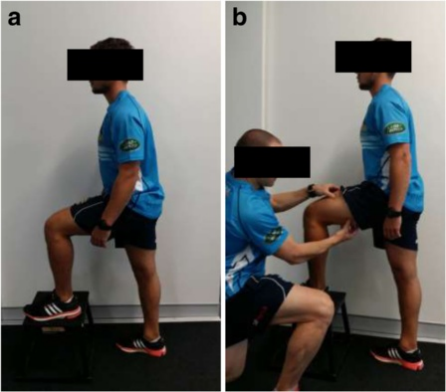
**a** Starting position for a ‘typical’ step up. **b** Starting position for the step up with deliberate co-activation – the participant is receiving tactile feedback on how to co-activate his quadriceps and hamstrings prior to commencement of the step-up. NB: Both persons in this figure gave both verbal and written consent to have their images published

CT data was collected from each participant’s self-reported dominant leg at 0.5 mm slice intervals on an Aquilion 16 (Toshiba, Tokyo, Japan) 150 mm above and below the knee joint. Then, participants performed a ‘typical’ step-up onto a 30 cm box under fluoroscopy (Axiom Artis MP, Siemens, Munich, Germany) while muscle activity was measured using an eight-channel telemetry EMG system (Mega Electronics, Kuopia, Finland) from four muscles (vastus lateralis, vastus medialis, biceps femoris long head, and semimembranosus). Fluoroscopy was performed in the sagittal plane. The step-up procedure was then repeated following training to increase hamstring-quadriceps co-activation. In order to increase co-activation tactile stimulation was applied participants’ quadriceps and hamstrings prior to them performing the ‘deliberate co-activation step-up’ (see Fig. 1; note both persons in this figure gave written and verbal consent to have their images published). They were then instructed to contract the muscles the experimenter was touching and hold that contraction as best they could for the duration of the step-up. Visual inspection, by the experimenter, of the raw EMG trace for the step-up with deliberation co-activation confirmed increased muscle activation relative to the ‘typical’ step-up. Participants were given as many practice trials they wanted on the deliberate co-activation step-up prior to performing the task under fluoroscopy however no participant took longer than five minutes to learn the task.

A 4-D model of the motion of the femur and tibia was created using an algorithm which produces a digitally reconstructed radiograph from CT data and then filters it to construct an edge-enhanced image. It was then registered to an edge-enhanced version of each fluoroscopy frame using gradient-descent based image registration as described elsewhere [6–8]. Error associated with this CT-fluoroscopy image registration technique is a standard deviation of 0.38 mm for in-plane translations and 0.42 degrees for rotations [8].

#### Kinematic analysis

**Fig. 2:**
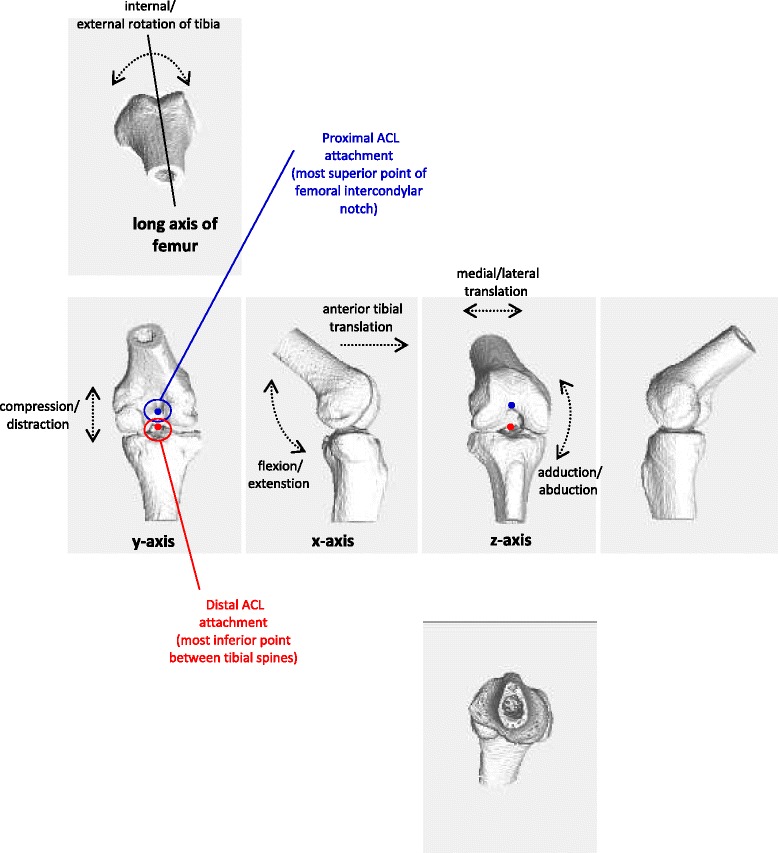
Example of typical CT-fluoroscopy image registered output for a step up with descriptions of how knee joint motion was measured. ACL length was measured as distance between the ACL attachments. Change in ACL length was considered the change those attachments moved relative to each other. Maximum knee joint translations and rotations, and ACL elongation was maximum change relative to starting position

Anterior-posterior movement (e.g. flexion and ATT) was measured on the x-axis, superior-inferior movement on the y-axis (e.g. compression/distraction), and medial-lateral movement on the z-axis (e.g. medial translation, abduction). The long axis of the femur provided the reference for rotation co-ordinates for the tibia. ACL attachments were defined according the method used by Grood and Suntay [9]; the proximal attachment was assumed to be the most superior point of the intercondylar notch of the femur and the distal attachment was assumed at the most inferior point between tibial plateau spines. ACL length was therefore taken to be the distance between these points and the change in ACL length equated to the change in distance between those points as they moved relative to each other. Maximum knee joint translations, knee joint rotations and ACL elongation were recorded as the maximum change relative to the first measurement. An example of a typical 4-D model with descriptions of how the kinematic analysis was performed can be seen in Fig. 2.

#### EMG collection and analysis

Care was taken to avoid crosstalk; following skin preparation, monopolar Ag-AgCl disc surface electrodes with a 2 cm radius (Ambu, Denmark) were placed at the approximate center of each muscle belly with a minimum of 1 cm separation in accordance with guidelines outlined by the Surface Electromyography for the Non-Invasive Assessment of Muscles (SENIAM) project [10]. The EMG signal was recorded by telemetry then converted from analogue to digital using an A/D converter (National Instruments NIUSM-6210, NSW, Australia) with a preamplifier gain of 305. A band-pass filter 12–450 Hz and a sampling rate of 1000 Hz with a common mode rejection ratio of 60 dB was applied. The signal was amplified using double differential amplifiers and subsequently recorded using Megawin software (Mega Electronics, Kuopia, Finland). It was then visually checked for artefacts before being exported to Microsoft Excel where a root mean squared (RMS) filter was applied at a non-overlapping moving window length of 20 ms. Peak RMS EMG was recorded for each muscle for both step-ups. Electrode removal did not occur between step-up conditions.

A co-activation index, which is the ratio of peak RMS EMG for antagonistic to agonistic muscle activity, was calculated for the medial hamstring and quadriceps muscles (semimembranosus-vastus medialis), the lateral hamstring and quadriceps muscles (biceps femoris-vastus lateralis), and the medial and lateral hamstring muscles (semimembranosus-biceps femoris) for both step-up conditions. Co-activation index for the medial and lateral quadriceps was not calculated because data showed that for the step-up with deliberate co-activation muscle activity was predominantly from the hamstrings not quadriceps, therefore we were only interested in the role of the hamstring muscles in modulating ACL elongation. To remain consistent with other work, extensor muscle activation was always the denominator for the hamstring-quadriceps co-activation indices [11] . For the purpose of consistency and ease of analysis, and because the denominator remained consistent for our flexor-extensor co-activation indices, the denominator was always the lateral hamstring for our medial-lateral hamstring co-activation index. Therefore, less valgus and knee rotation was expected for a smaller semimembranosus-biceps femoris co-activation index. Finally, timing of peak RMS EMG for each muscle relative to their co-activation index antagonist muscle was established for both step-up conditions to ensure the co-activation index was a true reflection of motor unit recruitment occurring at approximately the same time either side of the joint. Comparisons of co-activation between step-up conditions were based on no electrode removal.

### Statistical analysis

Due to the small sample size only descriptive statistics were presented for comparison of means between step-up conditions for all EMG and kinematic data. However, data for both step-up conditions was pooled and a Pearson’s correlation was used to test for relationships between ACL elongation and kinematic data, and ACL elongation with co-activation indices. Significance was set at α ≤ 0.05.

## Results

**Fig. 3:**
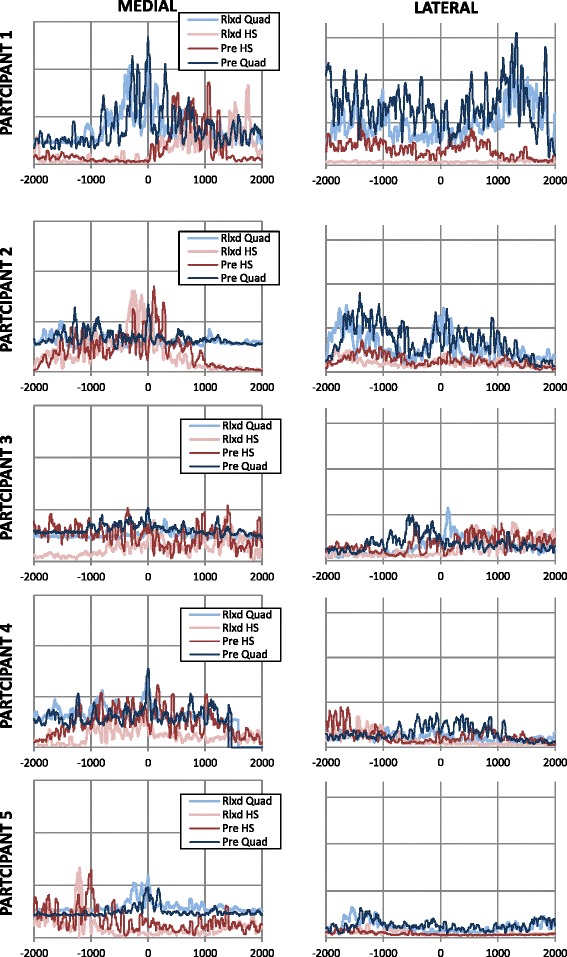
EMG Traces for medial quadriceps and hamstrings (vastus medialis and semi-membranosus respectively), and lateral quadriceps and hamstrings (vastus lateralis and biceps femoris respectively). NB: Quad = quadricep; HS = hamstring; Rlxd = relaxed and observed on first step-up; Pre = pre-activated and observed on step-up with deliberate co-activation. 0 = timing of peak vastus medialis activation for all graphs (msec)

The step-up with deliberate co-activation resulted in greater activation of the hamstrings, greater co-activation indices for semimembranosus-vastus medialis and biceps femoris-vastus lateralis, and a smaller co-activation index for semimembranosus-biceps (Fig. 3 and Table 1). Furthermore, the period of time between peak activation for each muscle in each co-activation index was smaller (Fig. 3 and Table 2).

**Table 1:** Maximum absolute muscle activation (RMS EMG), and co-activation index for both step-up conditions (mean ± SD)

	Vastus medialis RMS EMG (mV)	Vastus lateralis RMS EMG (mV)	Semimembranosus RMS EMG (mV)	Biceps femoris RMS EMG (mV)	Co-activation index		
					Semimembranosus: Vastus Medialis activation index (mV)	Biceps Femoris: Vastus Lateralis Co-activation index (mV)	Semimembranosus: Biceps Femoris Co-activation index (mV)
Low level co-activation step-up	280.67 ± 111.10	438.00 ± 347.63	248.84 ± 84.14	159.07 ± 82.14	0.94 ± 0.33	0.59 ± 0.47	2.28 ± 1.84
95 % confident interval							
Upper limit	378.05	742.71	322.60	231.07	1.23	1.00	3.89
Lower limit	183.29	133.29	175.09	87.07	0.65	0.17	0.67
Step-up with deliberate co-activation	302.08 ± 137.74	430.48 ± 279.11	346.04 ± 143.47	311.70 ± 190.18	1.16 ± 0.14	0.88 ± 0.78	1.50 ± 1.02
95 % confidence interval							
Upper limit	422.82	675.13	471.79	478.40	1.29	1.56	2.40
Lower limit	181.35	185.84	220.28	145.01	1.04	0.20	0.60

NB: RMS = Root Mean Square. Co-activation index values are hamstrings divided by quadriceps or medial hamstrings divided by lateral hamstrings

**Table 2:** Difference in timing of peak activation for each muscle in the co-activation indices (mean ± SD)

	Vastus Medialis – Semimembranosus (msec)	Vastus Lateralis – Biceps Femoris (msec)	Semimembranosus – Biceps Femoris (msec)
Low level co-activation step-up	0.55 ± 2.48	−2.53 ± 5.75	−1.57 ± 3.27
95% Confident Interval			
Upper Limit	3.63	4.62	2.49
Lower Limit	−2.53	−9.67	−5.63
Step-up with deliberate co-activation	−0.18 ± 8.52	−1.96 ± 11.04	−0.70 ± 5.34
95% Confidence Interval			
Upper Limit	10.39	11.74	5.93
Lower Limit	−10.76	−15.67	−7.32

NB: Values are hamstring prior to quadriceps or lateral hamstrings before medial hamstrings

Stepping-up with deliberate co-activation consistently resulted in reduced kinematic excursions and decreased elongation of the ACL during the step-up task (Table 3). Analysis of pooled data showed that as the ACL lengthened the knee abducted (*r* = 0.91; *p* < 0.001), distracted (*r* = −0.70; *p* = 0.02 for relationship between knee joint compression and ACL elongation) and anteriorly translated (*r* = 0.52; *p* = 0.01) (Table 4). However, no significant relationship was demonstrated between ACL elongation and internal rotation (*r* = 0.07; p = 0.85), or for ACL elongation and medial translation (*r* = 0.44; *p* = 0.21).

**Table 3:** Mean maximal change in knee joint kinematics from start position for both step-up conditions, including internal rotation, knee abduction, medial shift, joint distraction, anterior tibial translation and ACL length (mean ± SD)

	Internal rotation (degrees)	Knee abduction (degrees)	Medial translation (mm)	Joint distraction (mm)	Anterior tibial translation (mm)	Change in ACL length (mm)
Low level co-activation step-up	-11.54 ± 3.16	15.73 ± 2.25	9.78 ± 4.00	-20.55 ± 2.57	2.67 ± 1.48	15.73 ± 2.25
95 % confident interval						
Upper limit	-7.61	18.52	14.75	-17.36	4.50	18.52
Lower limit	-15.47	12.94	4.82	-23.74	0.83	12.94
Step-up with deliberate co-activation	-10.94 ± 4.26	13.92 ± 1.94	7.78 ± 3.60	-20.42 ± 2.51	1.22 ± 0.59	13.92 ± 1.94
95 % confidence interval						
Upper Limit	-5.68	16.33	12.25	-17.31	1.95	16.33
Lower Limit	-16.27	11.52	3.31	-23.54	0.50	11.52

**Table 4:** Relationships between ACL Length and Internal Rotation, Knee Abduction, Medial Shift, Joint Compression and Anterior Tibial Translation

	Internal rotation (degrees)	Knee abduction (degrees)	Medial translation (mm)	Joint compression (mm)	Anterior tibial translation (mm)
Change in ACL Length (mm)	0.07	0.91	0.44	-0.70	0.52
p-value	0.85	≤0.001	0.21	0.02	0.01

NB: α = 0.05

**Fig. 4:**
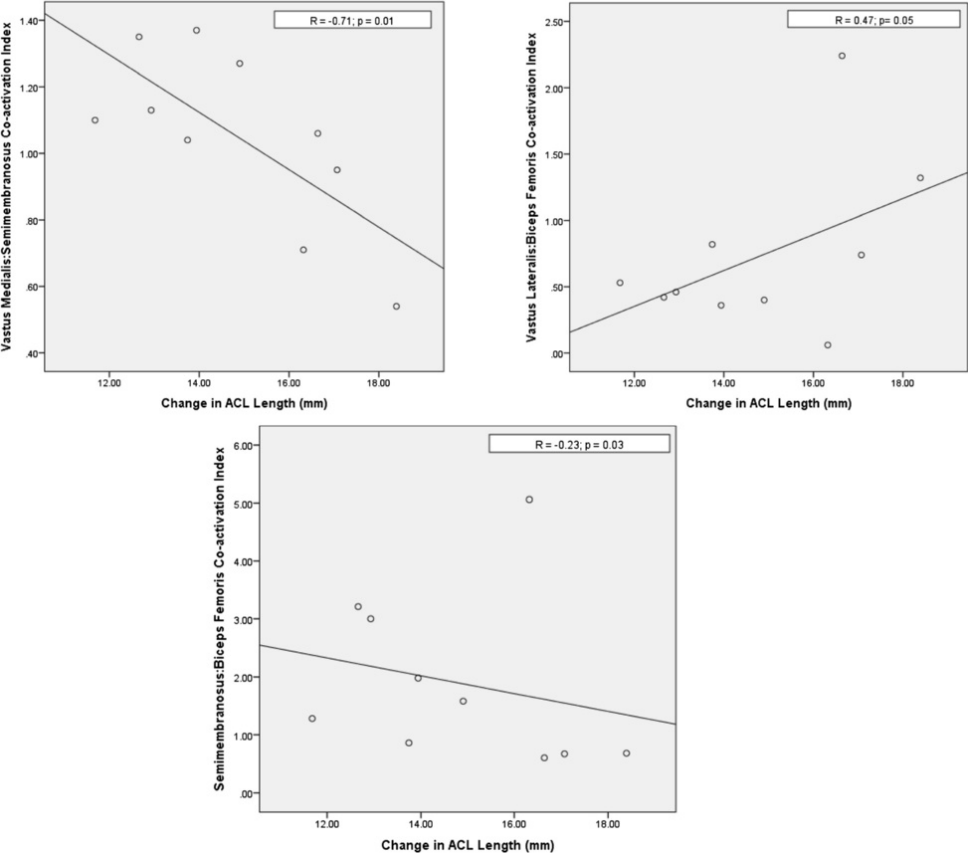
Relationships between EMG co-activation indices illustrating that net hamstring activation and medial, not lateral, co-activation is related to shorter ACL length (mm)

Stronger medial hamstring-quadriceps co-activation, demonstrated by a higher semimembranosus-vastus medialis co-activation index, resulted in a shorter ACL (*r* = −0.71; *p* = 0.01) (Fig. 4). With stronger lateral hamstring-quadriceps co-activation, demonstrated by biceps femoris-vastus lateralis co-activation index, the ACL lengthened (*r* = 0.47; *p* = 0.05). Finally, the ratio of medial to lateral hamstrings activity decreased as the ACL lengthened (*r* = −0.23; *p* = 0.03) meaning that increased medial hamstrings activity was associated with a shorter ACL.

## Discussion

The purpose of this pilot study was to investigate whether hamstring-quadriceps co-activation altered knee joint motion and limited ACL elongation during a step-up task. Although preliminary, the results of this study indicate that increasing co-activation of select hamstring and quadriceps muscles during a step-up task appears to reduce knee joint rotation, abduction, translation and distraction. Not surprisingly therefore, a lesser amount of ACL elongation was observed during the step-up with deliberate co-activation.

Change in ACL length correlated with co-activation of both lateral and medial muscle groups. However, because ACL elongation was positively correlated to the biceps femoris-vastus lateralis co-activation index and inversely correlated to the semimembranosus-vastus medialis co-activation index it is likely that medial hamstring-quadriceps co-activation, not lateral, is associated with smaller ACL elongation. This finding suggests that net co-activation of the hamstrings and quadriceps may reduce ACL elongation provided that the proportion of medial hamstring-quadriceps co-activation exceeds lateral. This hypothesis is supported by our finding that knee abduction, a movement influenced by vastus lateralis and biceps femoris [12–14], was positively correlated with ACL elongation (Table 4). These findings are meaningful when one considers that current knee reconstruction techniques involve harvesting medial hamstring tendon for ACL grafts.

The study presented in this paper is novel because this is the first time knee joint kinematics and active laxity, in the form of knee joint translations, have been measured *in-vivo* directly from bone. The methodology has a proven high degree of accuracy [8] and has the advantage of allowing concurrent EMG measurement of muscle activity. Previous studies have lacked accuracy because they have only been able to infer active laxity measurement from measures of patella tendon angle without concurrent measurements of muscle activity [2], or have had to extrapolate from *in-vitro* experiments [5].

The reductions in ACL length and ATT associated with co-activation are small but the implications are significant. Our research showed that, for a seemingly basic task such as a step-up, ATT and ACL elongation can be reduced by approximately 1.2 mm and 2.0 mm (respectively) with deliberate co-activation of select hamstring-quadriceps muscles. Previous studies have indicated that failure of the ACL is associated with relatively small changes in ACL length; an *in-vivo* study of passive laxity after ACL injury indicated that left-right 3.0 mm differences in passive laxity on an anterior drawer test is indicative of ACL injury [15]. Cadaveric studies have shown a difference in ATT of approximately 7.0 mm pre and post ACL rupture [16] and primate model research showed the ACL began to fail when stretched by just 5.4 mm and this was exacerbated by the speed at which strain was applied [17]. Good comparisons between animal models and human ACL elongation patterns have been established [18]. Therefore, in view of the small length changes which appear to be required for failure of the ACL, the changes in ACL elongation detected in this study after very simple co-activation training should be considered clinically meaningful in terms of injury prevention and rehabilitation.

The potential for modulation of ACL elongation via neuromuscular training of the medial hamstring muscles is an important implication arising from of this study. There is a possibility that over activity of the lateral hamstrings and quadriceps could put the ACL at risk. This is of particular concern in the patient who has had an ACL repair using a medial hamstring graft given that muscle inhibition can persist for up to 12 months following a muscle strain injury [19]. Increased activity of the lateral hamstrings and quadriceps might ensue following trauma to the medial tendon and could be a contributing factor to the fact that history of ACL injury is a significant risk for ACL injury [1]. This theory is also supported by some opinion which has presented a good argument for prior hamstring injury being a risk factor for ACL injury [14]. However, some caution must be exercised when considering and interpreting these findings because increased co-activation of the medial hamstrings and quadriceps muscle may be associated with osteoarthritis of the knee [20, 21], particularly when one considers that people with prior ACL injury are at increased risk of developing osteoarthritis of the knee later in life [22, 23]. Furthermore, the relationship between the muscles is not necessarily closed, it could be synergistic [24]. Synergism is defined as the distribution of force among individual muscles to produce a given task [24, 25]. The role of each muscle in a given muscle group may be modulated by a synergistic muscle [26], and it is known that the central nervous system considers synergistic muscles as a functional unit as opposed to single motor units [27].

This study has a number of limitations. Firstly the cohort studied was small but as a pilot study the results are promising and, in our view, because of the clinical relevance of hamstring-quadriceps co-activation for ACL injury a larger study is justified despite financial and ethical considerations. Secondly, limitations surrounding EMG data collection were present. For instance, we did not quantify EMG cross-talk when measuring muscle activity. However, methods for measuring cross-talk, such as EMG signal cross-correlation, have been shown to be ineffective in identifying cross-talk [28]. Therefore the likelihood of cross-talk measurement occuring was simply minimized by collecting EMG data according to SENIAM guidelines and applying a double differential signal amplifier which has been shown effective in minimizing cross talk [29]. In addition to this, only peak absolute RMS EMG data was presented; it could be argued that peak RMS EMG normalized to maximum voluntary contraction should be presented as it describes better the magnitude of muscle activation. However, EMG was only used to confirm an increased level of co-activation of selected hamstring and quadriceps muscles for the step-up with deliberate co-activation. Given that the same electrodes were used on the same day on the same participants without removal between step-up conditions, and an increase in activity was seen for each muscle it can confidently be concluded that increased muscle activity was achieved for the step-up with deliberate co-activation. Furthermore, because the difference in timing of peak activation between muscles in each co-activation index reduced for the step-up with deliberate co-activation then we can state with confidence that a higher level of co-activation was achieved and not just increased activation of agonist and antagonist muscles occurring at significantly different time points. A third limitation is related to the statistical analysis for the comparison of means for EMG and kinematic data. We presented only descriptive statistics because the sample size was small. Parametric statistical analysis was not possible because the data did not satisfy the assumptions required for this type of analysis and a non-parametric analysis would likely return a type II error. A greater sample size would allow for statistical analysis for comparisons of means and is necessary to confirm our findings. Finally, timing of peak ACL elongation relative to hamstring-quadriceps co-activation, and muscle activity throughout the gait cycle was not reported. While we can confidently say that the step-up with deliberate co-activation resulted in a higher level of co-activation, we cannot be accurate about when this occurred. Unfortunately, however, it is not possible with currently available technology to synchronize EMG with the image registration technology described in this paper. Assumptions about muscle activation relative to commencement of movement have been well established elsewhere [24, 30–34] and therefore must be considered.

The results of this pilot study are promising. A future study powered for statistical examination and with some methodological improvements such as requiring participants to complete a more ecologically valid task relevant to ACL injury and enhancing EMG data collection is justified.

## Conclusion

This pilot study sought to examine the clinical assumption that hamstring-quadriceps co-activation results in constraining knee from excessive ATT and other kinematic excursions therefore protecting the ACL from elongation. Our preliminary results suggest that medial hamstring-quadriceps co-activation may constrain ACL elongation, however if lateral activation exceeds medial then ACL elongation might ensue. Although the results need confirmation with a larger study, the clinical implications are meaningful in terms of risk assessment and injury prevention.

## Abbreviations

ACL: Anterior cruciate ligament
EMG: Electromyography
ATT: Anterior tibial translation
CT: Computed Tomography
